# The EU endocrine disruptors’ regulation and the glyphosate controversy

**DOI:** 10.1016/j.toxrep.2021.05.013

**Published:** 2021-06-03

**Authors:** Paraskevi Kalofiri, Giorgos Balias, Fotios Tekos

**Affiliations:** a Harokopio University of Athens, Greece; b Department of Biochemistry-Biotechnology, University of Thessaly, Larissa, Greece

**Keywords:** Endocrine disruptors, EDC, EU regulation, Glyphosate, Pesticide legislation, Precautionary principle

## Abstract

Endocrine disruptors are compounds that alter the functioning of the endocrine system of humans and wildlife. A large number of chemicals have been identified as EDs and humans can be exposed to them through dietary and/or environmental exposure (air, water, soil). At international level, scientific discussion on the topic of EDs focuses on the issue of setting out the scientific criteria according to which the key properties of these substances that render them EDs are determined. Regulatory action in EU has been impacted by the aforementioned discussion and, in particular, Regulation 2017/2100 and Regulation 2018/605 have been issued. However, these scientific criteria do not constitute a complete framework for the detection of EDs and, therefore, their adoption does not entail a fully effective human health protection. Moreover, glyphosate-based herbicides (GBH), are the most widely used pesticides worldwide. The glyphosate controversy turned the spotlight on pesticide regulation in the EU. The disagreement between IARC and regulatory evaluations of EFSA/ECHA has received great attention of citizens, organizations and stakeholders, as a result of methodological differences in the evaluation of the available evidence have been identified. This paper outlines the glyphosate controversy, following an overview of the EU EDs Regulation and pesticide legislation.

## Highlights

• Endocrine disruptors are compounds that alter the functioning of the endocrine system of humans and wildlife.
• Regulation 2017/2100 and Regulation 2018/605.
• Glyphosate as Endocrine Disruptor.
• EU EDs Regulation and pesticide legislation.

## 1 Introduction

It is established that Endocrine Disruptors (EDs), constitute one of the most serious risks to human health [1] as they severely disrupt the endocrine system [2]. This risk is largely linked with, among other, the problem of identification of the various chemical substances contained in a wide range of man-made products [3] such as pesticides, biocides, cosmetics, plastics, paints, construction materials, and other items used daily. EDs also occur naturally such as in hormones and plant estrogens [4].

More specifically, it is acknowledged in the scientific community that certain chemical substances act upon the endocrine-hormonal system in such a way that they cause disruption and, therefore are capable of interfering with the processes of development both in humans and wildlife. Human exposure to EDs occurs through swallowing food, dust and water, through inhalation of air particles, through the skin, as well as through the maternal tract. Embryos, babies and children are the groups more susceptible to these substances [5].

To address the risk caused by Endocrine Disruptor Chemicals (EDs), EU has introduced a relatively complete legal framework. In particular, EU legislation regarding the chemical substances that may potentially function as EDs consists mainly of: Regulation 528/2012 [6] concerning biocides, Regulation 1107/2009 [7] concerning plant protection products, the REACH Regulation [8] 1907/2006 and Regulation 1223/2009 for cosmetic products [9].

At international level, scientific discussion on the topic of EDs focuses on the issue of setting out the scientific criteria according to which the key properties of these substances that render them EDs are determined. Regulatory action in EU has been impacted by the aforementioned discussion and, in particular, Regulation 2017/2^1^00 [10] and Regulation 2018/605 [11] have been issued. However, these scientific criteria do not constitute a complete framework for the detection of EDs and, therefore, their adoption does not entail a fully effective human health protection.

The impact on the endocrine and reproductive system, as a result of xenobiotics, is believed to be due to several factors that include mimicking of endogenous hormones, such as estrogens and androgens, antagonizing the effects of physiological endogenous hormones, altering the synthesis process and metabolism of natural hormones, and altering hormone receptor levels [12,13].

Therefore, EDs are defined as those substances that are capable of inflicting the hormonal system of living species and interfering with, as well as altering, physiological processes. The World Health Organization (WHO) has defined EDs as following: “*An endocrine disruptor is an exogenous substance or mixture that alters function(s) of the endocrine system and consequently causes adverse health effects in an intact organism, or its progeny, or (sub)populations”* [14]. WHO proceeded to make the above definition even more specific by exactly specifying the adverse effects [15]. Furthermore, in its report, EFSA expressed its total agreement with the above findings regarding the risk for human health and the environment, even clarifying some of the parameters that had emerged from the discussion [16].

More specifically, according to EFSA, EDs are substances that have adverse effects on living organisms (e.g. changes in morphology, physiology and development), while showing a causal relationship between these effects and the ED mode of action, which is to interfere with and act upon the endocrine system (thyroid gland, ovaries, etc.) [17].

During the last few decades, the increasing rate of occurrence for certain diseases excludes genetic factors as the only plausible explanation. Environmental and other non-genetic factors, including nutrition, viral diseases and exposure to chemical substances, must be taken seriously into account, however difficult their detection may be [18]. Despite these difficulties, certain examples of chemical substances that act as EDs have made apparent their correlation with adverse effects on health and the environment [19].

It is a constantly changing landscape, as some EDs have been banned for decades while others more recently, yet there are still significant differences among countries. There have also been historical examples of toxic leakage or contamination (e.g. PCB and dioxins) [20] that indicate a direct causal relationship between a chemical substance and the manifestation of endocrine or reproductive dysfunction in humans and wildlife. However, these isolated cases of exposure are not representative of the more usual and widespread persistent exposure to a wide mixture of chemical substances [21]. These complex mixtures enter the food chain and accumulate in animals that are higher in the food chain [22].

## 2 The EU EDs regulation

The term endocrine disruption was mentioned for the first time in 1992 in an attempt to provide explanations for several pathological conditions and anomalies observed in wildlife and humans - conditions that pertained to interference of substances with the endocrine system by means of mimicking behavior and other mechanisms, as previously mentioned [23].

In 1999, the European Commission published its strategy for EDs which described actions necessitated in the EU, including short-term (research, international collaboration), mid-term (trial methods) and long-term action (establishment of a special regulatory framework) with the ultimate goal to reduce exposure of the public to the lowest possible extent [24]. Since then, EU has adopted a series of regulatory measures with respect of the identification of EDs which concerns special areas such as water management [25], cosmetics [26], and chemical products [27].

According to the REACH Regulation, the ECHA has included substances that exhibit properties of endocrine system disruption on the list of chemical substances that are alarmingly worrying and require licensing [28]. Regulation 528/2012 and Regulation 1107/2009 are also of particular importance [29,30]. Biocides as well as plant protection products may entail several health risks for humans, animals and the environment due to their inherent properties and relative uses [31]. In view of these risks and in order to ensure a high level of protection for human health, wildlife and the environment while at the same time allowing the functioning of the internal market for such products, the two aforementioned regulations dictate that the active chemical substances used in plant protection products and biocides are approved at EU level - and only for a limited period of time - before they become available on the market.

Approval of these substances is provided only when these exhibit no adverse effect on human health and the environment [32]. Therefore, particularly dangerous substances are not licensed and these include active substances which *“are considered as having endocrine-disrupting properties that may cause adverse effects in humans”* [33] or *“have an inherent capacity to cause endocrine disrupting”* [34].

It should be noted that both Regulations stipulate that it is the Commission's duty to specify the scientific criteria regarding the characterization of a product as an ED, which pertains both to biocides and plant protection products [35].

## 3 The role of scientific knowledge in the decision-making regarding EDs

In compliance with the regulatory framework described above, the European Commission set out scientific criteria with respect of the specification of EDs properties. Therefore, the Commission is based on, and limited only to, facts and scientific data, and does not consider any other data particularly connected with the economic repercussions arising from the EDs regulation. As stated by the Commission, in order to characterize a product as ED, the only requirement is whether this chemical substance disrupts the endocrine system, regardless of the extent of the disruption [36].

The findings and conclusions of WHO have become accepted by virtually the entire international scientific community, as mentioned previously. It is now commonplace in science that there are three salient features that define a chemical substance as ED: hormonal function, adverse effects, and causal relationship between the two [37]. These characteristics are included in the Regulations for the setting out of criteria and apply to biocidal products (Regulation 2100/2017) [38] as well as plant protection products (Regulation 605/2018) [39].

Regulation 605/2018 sets out the scientific criteria as concerns the specification of EDs properties according to Regulation 1107/2009.^1^ Emphasis is laid on the fact that it is important to develop such scientific criteria in view of ensuring a high level of protection for human and animal health, as well as the environment [40].

More specifically, Regulation 605/2018 enshrines the definition given by WHO for EDCs as expressed in the reports of 2002 and 2009. It also points out that specification of endocrine disrupting properties should be based on evidence pertaining to humans and wildlife so that the recognition of known [41], as well as potential, substances with ED properties is possible [42]. To be more accurate, endocrine disrupting properties are defined in detail in the Regulation annex and, more specifically, the criteria are set out in articles *a* and *b* of that Regulation. Furthermore, the REACH Regulation [43] states the adverse effects on sexual function and fertility and development. Products that may entail such risks are considered to cause endocrine disruption and should not be placed on the market. It should be noted that the REACH Regulation includes clear provisions regarding EDs. In particular, according to article 57 of this Regulation, annex XIV must register various chemical substances that display hazardous properties including those of endocrine disruption [44].

## 4 Plant protection products

Approval procedures for plant protection products in order to be placed in the EU integral market are governed by Regulation 1107/2009 [45], which is considered a complete legislation regarding plant protection products. The Regulation requires that the assessment of an active plant protection substance should be *“independent, objective and transparent”* and also be conducted *“in the light of recent scientific and technical developments”* [46]. In addition to safeguarding agriculture in EU, the purpose of the Regulation is also *“to ensure a high level of protection of both human and animal health and the environment”* [47]. It is noteworthy that the provisions of the Regulation are underpinned by the precautionary principle, and indeed the Regulation makes a direct reference to it pointing out that *“The precautionary principle should be applied and this Regulation should ensure that industry demonstrates that substances or products produced or placed on the market do not have any harmful effect on human or animal health or any unacceptable effects on the environment”* [48].

Regulation 1107/2009 introduced for the first time criteria according to which the use of a chemical compound in any plant protection product is prohibited if this presents risk for human health (carcinogen, mutagen, toxic to reproductive system, endocrine disrupting) or the environment (persistent organic pollutant – POP, persistent, bioaccumulative and toxic substances – PBT, very persistent and very bioaccumulative substances – vPvB). In other words, the inherent risk in using a certain substance is sufficient reason not to place a product on the market. From a regulatory point of view, this means that a risk based approach has been selected [49]. However, Annex II of this Regulation introduces a deviation from the directive, or exception, in cases of *‘negligible exposure”*, in which substance levels are low enough to be safely considered negligible [50]. Therefore, regarding plant protection products, substances with endocrine disrupting properties cannot be approved unless human exposure to these under realistic conditions of recommended use is negligible [51]. More precisely, according to paragraph 3.8.2 (environment) of the same Annex, an active substance is approved only if *“it is not considered to have endocrine disrupting properties that may cause adverse effects on non-target organisms unless the exposure of non-target organisms to that active substance in a plant protection product under realistic proposed conditions of use is negligible”* [52,53]. Based on the above, the suggested approach in the directive seems to be making an exception to the rule introducing risk based assessment instead of strictly forbidding all types of use. Furthermore, the transition from negligible exposure to negligible risk is not clarified in the Regulation [54].

## 5 Glyphosate as ED

Glyphosate is a broad-spectrum active substance that has been used globally since its first application in 1974. Glyphosate is mixed with, and serves as a basis for, other chemical compounds known as ‘inert ingredients’. It is included in herbicide such as ‘Roundup®’ and ‘RangerPro®’ that are widely used in farm fields and in house gardens [55]. Wide use of glyphosate in agriculture has promoted the spread of high-tolerance resistant pests, which in turn has created the need for more frequent applications of the chemical and at higher concentrations. Humans may be exposed to the substance through various mechanisms such as food ingestion or water consumption both at work and the home environment [56]. Abusive usage of these compounds has led to adverse effects such as ground and river contamination, and residue accumulation in the food chain. At present, use of glyphosate as glyphosate-based herbicides(GBHs) is becoming increasingly widespread globally and the levels of environmental contamination indicate high concentrations of these compounds, with levels being significantly higher in countries or areas where agricultural activity is more prominent [57]. The first indications of mainly came from studies in the reproductive system of males. These endocrine disruptions are linked with glyphosate whether it is present as an isolated substance or the mixture in which it is contained [58].

In 2015, the International Agency for Research on Cancer (IARC) characterised glyphosate as “potentially carcinogenic” for humans [59]. However, in 2015, EFSA stated that it *“is unlikely to pose a carcinogenic hazard to humans”* [60]. Although EFSA does not deviate from its initial assessment, the new approval of the European Commission for glyphosate is more restrictive as it provides renewal only for a 5-year period instead of the usual 10-year. The European Commission also recommends a new set of preventive measures regarding its use (for example it recommends it not be used in public gardens and playgrounds) [61]. The US Environmental Protection Agency (EPA) also examined the substance in 2016 and, based on typical non-professional reports, arrived at the conclusion that it is not possible for it to be carcinogenic for humans [62].

Glyphosate has been thoroughly assessed by member states, the EFSA and the ECHA in order to assess the extent to which its use potentially causes adverse effects on human and animal health, and on the environment. In 2002, after such a risk assessment, glyphosate was approved for the first time according to the EU pesticides regulation. Before this assessment, it had been approved for use by members states according to national rules [63]. On November 27th 2017, the Board of Appeal concluded by majority in favour of the proposal of the European Commission for renewal of glyphosate use for a period of 5 years [64]. During the conference, modifications were made to the Commission Implementing Regulation. In June 2017, EFSA published an opinion regarding EU's assessment of glyphosate after allegations made in the so-called ‘Monsanto Papers’. The report requested from the European Commission describes EU normative framework regarding the submission of an open scientific bibliography for the assessment of active substances and explains the manner in which this bibliography will be considered by the state members of EU and experts of EFSA during assessment procedures [65]. In July 2017, upon request of the European Commission, EFSA and the ECHA replied to a letter sent by Professor Christopher Portier to president Juncker regarding the investigation and assessment of glyphosate carcinogenic properties [66]. In September 2017, the integrity of glyphosate risk assessment conducted by EU was disputed, especially as concerns the content of the assessment report submitted by EFSA to the German Federal Institute for Risk assessment (BfR) [67]. EFSA replied with an announcement in which it defended the credibility of EU risk assessment and pointed out that allegations were based on a misunderstanding of the procedure by peer assessors.

The contrasting evaluations of EFSA/ECHA and IARC can be explained by the different methodologies followed. While IARC examined both glyphosate as an active substance as well as the plant protection products in which it was contained (for example Roundup™), the assessment conducted by EU agencies on the other hand examined only glyphosate as a substance, on the grounds that members states are the ones authorized for the approval of any plant protection product that is placed on the market within their countries. Additionally, while IARC examined only published studies, EU agencies took into account studies submitted by those applying for glyphosate license [68]. Therefore, EFSA/ECHA and IARC followed distinctly different approaches of weighing the available data producing contrasting conclusions as a result.

A considerable number of scientists have attempted for more than 30 years to investigate and assess the role of glyphosate as an ED using in vitro, in vivo and epidemiological studies. However, despite the results obtained, there is no consensus regarding the repercussions and the dangers that glyphosate may entail for the human endocrine system [69]. Furthermore, the various effects that cause endocrine disruption triggered by the several products that contain glyphosate indicate that the results may be due to other ingredients in the product besides glyphosate, many of which are unknown [70]. This inconsistency in conclusions may possibly be due to the fact that certain studies are exempted from re-examination by EFSA and certain unpublished data is not included in the re-examination of EPA [71].

The controversy regarding the carcinogenic classification of glyphosate depends on various parameters, including the significance attributed to epidemiological studies in humans [72]. Following the EU approval of the active substance, products containing glyphosate are placed on the market, sold and used according to license procedures determined by each member state individually. Furthermore, concerning pesticides based on glyphosate, the final chemical composition of a product, as sold and used, has been found to be of higher toxicity than the stated toxicity of glyphosate isolated from the other ingredients (in vivo studies) [73]. However, the full composition of the final product is not controlled for long-term toxicity [74]. This oversight during trials before product placement on the market has resulted in the availability of pesticides that exhibit unacceptable toxicity profiles, the negative effect of which was revealed after they had been approved [75]. Omission of assessing the cumulative and synergistic effects of chemical substances constitutes violation of corresponding regulations. Regulation 1107/2009 states that residue of any pesticide, which may contain sub-ingredient residue, should not have adverse effects on human or animal health, *“taking into account known cumulative and synergistic effects”* [76]. In 2019, EFSA published guidance on methods for assessing hazards to both human and animal health stemming from combined exposure to multiple chemical substances. However, other national agencies did not impose tighter restrictions – on the contrary, extended the license for glyphosate use [77].

According to P. Mu∼noz, et al., results from certain epidemiological studies on women that had been exposed to glyphosate indicate an increased risk of missed miscarriage and reduction in fertility. Furthermore, epidemiological reports show a correlation between exposure to glyphosate and the high risk of adverse reproductive effects and genetic anomalies in progeny. Therefore, it can be concluded that glyphosate acts as an ED that modifies hormonal activity and causes defects in the reproductive process and in progeny [78]. Also, according to Paola Ingaramo et al., there is a correlation between endocrine activity caused by glyphosate/GBHs and the adverse effects on female reproduction. Several studies have shown the presence of endocrine disruption, whether this may be caused by glyphosate alone or by products that this substance is contained in, depending on substance levels and exposure time [79]. It should be stressed at this point that certain products are protected by patent laws, and thus, their ingredients are unknown. For the precise reason that there is a difference between the measured effects of an active substance in the laboratory and those observed in the interactive environments in which the substance is used, risk regulators should review their procedures so that assessment methods take into account realistic conditions [80].

## 6 Real life exposure

**Fig. 1:**
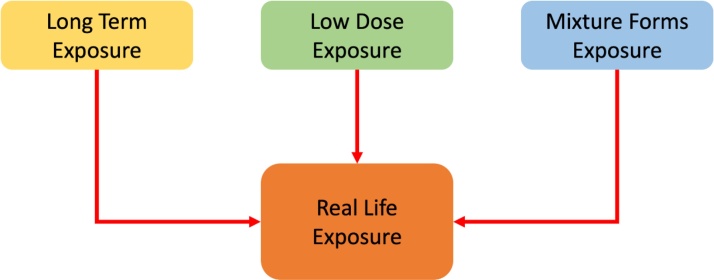
Modern parameters that affect real life exposure in chemicals contributing to detrimental impacts.

A big issue that has not been extensively researched so far is the deep understanding of the effects of various chemicals exposure, such as EDs in small concentrations below NOAEL on human’s daily routine. Modern everyday life brings us in contact with a plethora of substances via different routes of exposure. Although they are in very small quantities, their combination and constant contact may lead to detrimental consequences [81]. This continuous contact for long periods of time, perhaps throughout the life of the person, contributes to the aggravation of various factors as well as to the causing of adverse pathological conditions. Specifically, many biomonitoring studies are describing the interrelation between long term exposure to several chemicals in doses below the regulatory limits with several pathologies like obesity [82,83], cardiovascular disease [84] and diabetes [85]. Safety doses levels are chosen after the documentation of studies evaluating single chemical exposures. Therefore, the combined real life exposure scenario could lead to synergistic effects that have not yet fully elucidated [86] (Fig. 1).

Such approaches are simulating the real life exposure and raise important safety issues that will readjust regulatory framework [[87], [88], [89]]. Actually, recently new studies have investigated the impact of long term low dose regimen of exposure to a combination of chemicals that include the ED glyphosate [[90], [91], [92], [93]]. In these studies a mixture of 13 chemicals were administered in rats in three doses schemes below NOAEL for 6, 12 and 18 months. Results indicate statistical significant alterations in redox related and biochemical parameters proposing the importance of investigating combinational exposure in xenobiotics that we come in contact with on a daily basis. Such phenomena are of utmost importance for regulatory bodies since humans usually are exposed in mixtures of chemicals from different sources in their routine. Hazard and risk assessment in our modern world will be re-organized after the investigation of such approaches that identify early effects of xenobiotic exposure and will further contribute to understand the controversies found in previous literature [94].

## 7 Lack of transparency

Transparency is an important aspect regarding this issue. In particular, lack of transparency becomes a severe problem when public authorities with insufficient resources are burdened with the role of assessing the huge volume of information generated by the industry, and must produce results that are complete, accurate and reliable [95]. It is worth mentioning that the story of glyphosate provoked one of the most acute crises in EU Risk Regulation at least during the last decade. The extent and intensity of the social, political, scientific and legal conflict it caused were unprecedented [96]. Of course, controversies regarding the active substance are deeply rooted in the conflicts that characterize EU Risk Regulation in general, and more specifically, the EU Food Regulation and Governance [97].

A serious aspect of the problem is that only rarely, if ever, are industrial studies published. In particular, the full reports of toxicity studies on animals provided by those requesting approval have not been made public. On the grounds of commercial secrecy and confidentiality, Regulation 1107/2009 allows industries to withhold study results and, therefore, these cannot be evaluated by independent experts or the public. Thus, protection of commercial interest creates obstacles that are difficult, if not impossible, to overcome during the current period as well as after 2021 [98].^2^ Another thing is that the register procedure for industrial studies allows the collection of a certain type of information, usually positive for the parties with a commercial interest, enabling industries not to publish unfavorable results [99,100]. Ultimately, this only serves companies as they may claim their products are safe [101].

At this point it should be noted that pesticide use in EU remains at a high level despite the targets that were set by the 7th Environment Action Plan of EU regarding harmless and sustainable pesticide use until the year 2020 [102], and despite the fact that, in the case of glyphosate, epidemiological and scientific data show a highly possible carcinogenic action [103,104].

Conflict of interest among those involved in glyphosate assessment in the EU in 2002 and 2017 is yet another dimension of the problem [105]. For instance, Roland Solecki, head of the Department Pesticides Safety of BfR, was charged in 2017 with the task of assessing glyphosate and its effects on health while, at the same time, being a long-standing associate of the International Life Sciences Institute (ILSI), an industry-funded organization [106].

It also seems that Monsanto had already known about the carcinogenic effect of the substance since 1999 and made an attempt to prevent the scientific work by distorting data that constituted evidence for this risk. According to these documents, Monsanto funded scientific research the ultimate goal of which would be to present glyphosate as a non-carcinogenic substance [107,108]. Additionally, according to sources [109], tens of pages from the risk assessment report conducted by EFSA on glyphosate are identical to excerpts from the study submitted by Monsanto for approval. Under these circumstances, on January 15th 2019, the Administrative Court in Lyon, France annulled the decision by which Roundup Pro 360, containing glyphosate, would be made available in the French market. Furthermore, on the 2nd of July 2019, Austria became the first European country that banned the use of all products containing glyphosate [110]. In view of all these issues, efforts for reformation of the general legislation on food are made while imposing, among other, more ambitious models of transparency [111].

## 8 Conclusion

The EU legislation does not ensure an effective way of dealing with endocrine disruptors. This is, especially due to the fact that no assessment so far has covered all the different aspects of ED behavior [112]. The glyphosate case has revealed that discrepancies in the implementation of EU pesticides regulation are perpetual and not isolated incidents. Adverse effects due to the exposure to glyphosate, whether pure substance or products that contain it as an ingredient, must be taken seriously into account by regulators in order to better determine and set out criteria for the safe use of this substance, and to forge a strategy for the prevention of damage to health and the environment based on the precautionary principle [113].

## Conflicts of interest

The authors declare no conflicts of interest.

## Footnotes

1 We will restrict ourselves to this Regulation only as Regulation 2017/2100 includes the same criteria with minor differentiation.

2 According to the new legal framework (as modified by Regulation 2019/1381) that will be in effect from 27 March 2021, publishing of industrial reports is a requirement. By exception, certain information contained in industrial studies pertaining to the Regulation 1107/2009 for pesticides (which includes the full composition of pesticide products) may remain confidential if the applicant for license adequately justifies his request for confidentiality by presenting verifiable evidence that disclosure could lead to considerable damage to any vested interests. However, it is important that even sensitive information must be disclosed if it is related to release of substances into the environment but only upon third-party request towards EU institutional body or a member state.
